# Diagnostic delay in monogenic disease: A scoping review

**DOI:** 10.1016/j.gim.2024.101074

**Published:** 2024-01-17

**Authors:** Rory J. Tinker, Miles Fisher, Alex F. Gimeno, Kayce Gill, Camille Ivey, Josh F. Peterson, Lisa Bastarache

**Affiliations:** 1 Division of Medical Genetics and Genomic Medicine, Vanderbilt University Medical Center, Nashville, TN; 2 Vanderbilt University Medical Center, Department of Child Neurology, Nashville, TN; 3 Vanderbilt University School of Medicine, Nashville, TN; 4 Annette and Irwin Eskind Family Biomedical Library and Learning Center, Vanderbilt University, Nashville, TN; 5 Department of Biomedical Informatics, Vanderbilt University Medical Center, Nashville, TN; 6 Department of Medicine, Vanderbilt University Medical Center, Nashville, TN

**Keywords:** Diagnostic delay, Genetic disease, Informatics, Rare disease, Scoping review

## Abstract

**Purpose:**

Diagnostic delay in monogenic disease is reportedly common. We conducted a scoping review investigating variability in study design, results, and conclusions.

**Methods:**

We searched the academic literature on January 17, 2023, for original peer reviewed journals and conference articles that quantified diagnostic delay in monogenic disease. We abstracted the reported diagnostic delay, relevant study design features, and definitions.

**Results:**

Our search identified 259 articles quantifying diagnostic delay in 111 distinct monogenetic diseases. Median reported diagnostic delay for all studies collectively in monogenetic diseases was 5.0 years (IQR 2-10). There was major variation in the reported delay within individual monogenetic diseases. Shorter delay was associated with disorders of childhood metabolism, immunity, and development. The majority (67.6%) of articles that studied delay reported an improvement with calendar time. Study design and definitions of delay were highly heterogenous. Three gaps were identified: (1) no studies were conducted in the least developed countries, (2) delay has not been studied for the majority of known, or (3) most prevalent genetic diseases.

**Conclusion:**

Heterogenous study design and definitions of diagnostic delay inhibit comparison across studies. Future efforts should focus on standardizing delay measurements, while expanding the research to low-income countries.

## Introduction

Genetic diseases are individually rare and may be difficult to diagnose, resulting in a significant delay between the time when a patient experiences their first symptom and receiving their diagnosis, a time interval commonly referred to as diagnostic delay.^1,2^ Although diagnostic delay has been studied for a wide variety of diagnoses (eg, rare cancers and rheumatological disorders), it is particularly important in genetic disease, where it has been implicated in excess morbidity, mortality, and anxiety for individuals with undiagnosed genetic disease.^1,3,4^ It results in a lengthy diagnostic odyssey in which an individual is subject to multiple ineffective diagnostic tests. Addressing diagnostic delay is of increasing importance given the emergence of new treatments and preventative strategies that are transforming clinical genetics from a diagnostic to interventional specialty. Consequently, there is broad interest in improving the quality and timeliness of genetic diagnosis. One step toward this goal is to measure diagnostic delay, which varies across diseases, populations, and clinical settings. To that end, many articles have been published that attempt to characterize and measure the extent and length of diagnostic delay for a variety of genetic diseases.^5-9^

Diagnostic delay is defined as the time interval between the onset of symptoms and a confirmed diagnosis of disease.^10^ Other synonymous terms in the literature include diagnostic time lag.^11^ Although this definition is widely accepted, there is a lack of consensus on how best to measure this interval.^12,13^ The primary area of ambiguity is related to the precise definition of the “symptom onset” time point.^14-16^ To assess this time point, one must first define what qualifies as a symptom for a genetic disease.^17^ Each genetic disease presents with different symptoms, some of them more common in the unaffected population then the genetic disease (eg, rash, fever, and cough).^18^ When we ascribe these common symptoms as the onset of genetic disease, are we certain that these are associated with the genetic disease? Furthermore, how do we define the symptoms of genetic disease in a world of phenotypic expansion and variable heterogeneity?^19^ The notion of symptom onset also implies a perspective. Is the time point defined as when a symptom is perceived by the clinician or by the patient themselves? Should the symptom onset point be on the first day of a symptom’s presentations or the first point at which the disease could have been diagnosed. Because a patient may delay seeking medical care of a symptom—either by choice or because of lack of access—these 2 perspectives may result in significantly different estimates. Furthermore, how do we define diagnosis in genetics? Is it at the point of clinical suspicion or at the point of genetic, biochemical, or clinical diagnosis.^20,21^ It is also unknown how population studied (eg, which country the study is conducted in), the setting it was conducted in (eg, in an academic medical center), and the ascertainment method (eg, chart review or questionnaire) to patient could affect diagnostic delay.

Given this methodological variation in measuring diagnostic delay, the purpose of the current scoping review is to systematically search and synthesize the literature on diagnostic delay in genetic disease to assess how diagnostic delay is measured in the literature. We will then aim to identify the following: (1) the methodology of how diagnostic delay is measured, (2) the differences and commonalities in study designs, and (3) how rates and study methods are changing with time.

## Materials and Methods

This scoping review adheres to the Preferred Reporting Items for Systematic Reviews and Meta-Analyses extension for scoping reviews. These guidelines for scoping reviews were published in 2018 and contain 20 essential reporting items and 2 optional items to include when completing a scoping review.^22^ A pilot search to identify relevant keywords and appropriate subject headings was conducted by a health sciences librarian (CI) and a content expert (RJT). The final search strategies were developed by a health sciences librarian (KDG) and refined through discussion with the content expert (R.J.T.). A comprehensive search was conducted in Embase (Elsevier), PubMed, Scopus, and Web of Science on January 17, 2023. The database search strategies are available in Supplemental Figure 1. The focus of the search strategies was to identify peer reviewed studies written in English that discussed diagnostic delays in genetic diseases. Exclusion and inclusion criteria are available in Supplemental Table 1.

Abstract and full text screening were performed using Covidence (Covidence systematic review software, Veritas Health Innovation), a web-based software platform for various types of reviews. Two content experts (R.J.T. and M.F.) independently screened all studies and met to discuss disagreements and reach consensus. This process was repeated for full text review of the included studies. See Figure 2 for the PRISMA-ScR flowchart. Two content experts (R.J.T. and L.B.) independently extracted the data using a Microsoft Excel spreadsheet. To standardize the recording of the length of diagnostic delay we recorded diagnostic delay from the median value when individual studies reported delay as both mean and median.. Consensus was obtained through discussion between the content experts. The extracted information related to the settings and methods in diagnostic delay and can found in full in Supplemental File 1.

To present the results, we prepared an overview of all results regarding the data items. We conducted descriptive statics on the data items using R and generated figures using R and Microsoft Excel 2019. Comparisons between numerical data was conducted using unpaired *t* tests. Data were assessed for normality and parametric data were assessed using a student *t* test, whereas nonparametric data were assessed using Mann–Whitney *U* test. Information regarding the economic developmental status of countries (extracted categorical variables developed country, developing country, or least developed country) was obtained from the Office of the High Representative for the Least Developed Countries at the United Nations and The United Nations Economic Statistical Annex.^23,24^ Data on the prevalence of genetic disorders were obtained from Orphanet annual report on the “Prevalence and incidence of rare diseases.”^25^ Diseases were annotated as early onset (reported age of onset before the age of 5) or late onset (after the age of 5) by a clinical geneticist (R.J.T.). These designations were conducted using disease descriptions from Online Mendelian Inheritance in Man (OMIM), Orphanet, and Genereview.^25-27^

## Results

### Search results

**Figure 1:**
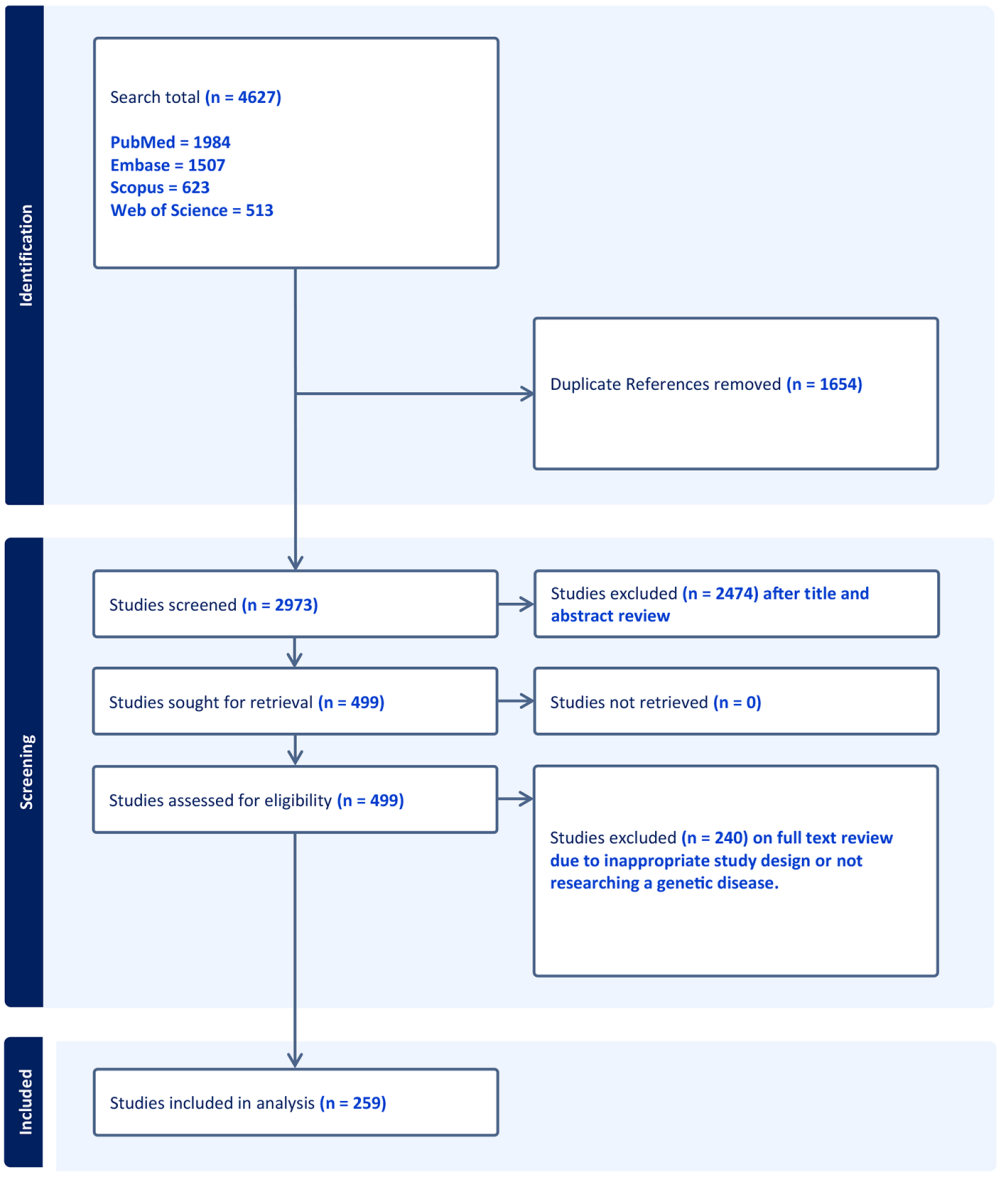
A flow chart demonstrating the inclusion and exclusion of studies in our literature search.

Our search strategy identified 4627 articles in our 4 searched databases (see Figure 1). When duplicate articles were removed (*N* = 1654), 2973 articles were available for title and abstract screening. In the first title and abstract screening step, 2474 articles were excluded, and 499 articles were considered relevant. After assessing the eligibility of the remaining 499 articles in title screening, 240 articles were excluded because they did not fit our inclusion criteria. This resulted in 259 articles for analysis. Of the 259 articles, 203 reported diagnostic delay as a single value (eg. diagnostic delay of 5 years for cystic fibrosis). Fifty-six articles reported diagnostic delay as stratified sub analysis (eg, by phenotype or genotype) or as a range and not as a collective value.

### Diagnostic delay research is increasing with time

**Figure 2:**
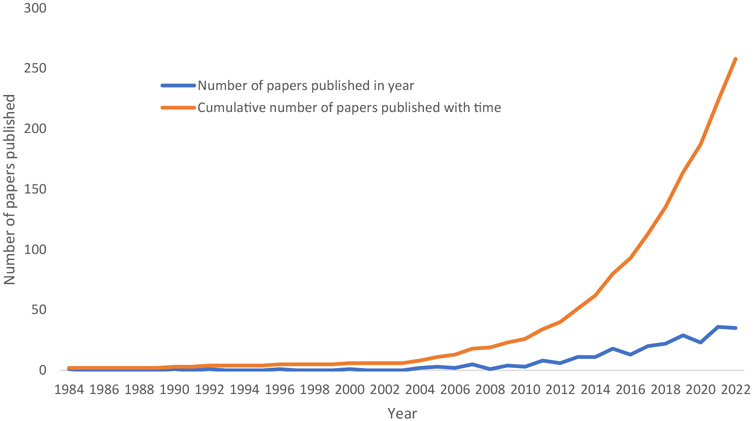
A cumulative line graph demonstrating the yearly number and cumulative total of studied reporting diagnostic delay between 1983 and 2022.

The number of published diagnostic delay studies were found to increase with publication year (see Figure 2). The first study reporting diagnostic delay was in 1983 with the latest published in 2023. 236 of the 259 studies were published since 2010.

### The most common genetic diseases in the diagnostic delay literature are not the most prevalent genetic diseases

The 259 articles included in the analysis measured diagnostic delay for 111 monogenic or groups of monogenic genetic diseases (see Table 1, Supplemental Table 2). The most common disease studied was hereditary angioedema (*N* = 26), familial Mediterranean fever (*N* = 16), and inborn errors of immunity (*N* = 12). When compared with the most prevalent 20 genetic diseases reported by Orphanet, there was no overlap with the analysis set, indicating that the most prevalent disorders are understudied with respect to diagnostic delay literature (see Supplemental Table 3).^25^

### There is variation in the reporting of diagnosis within the same diseases

**Figure 3:**
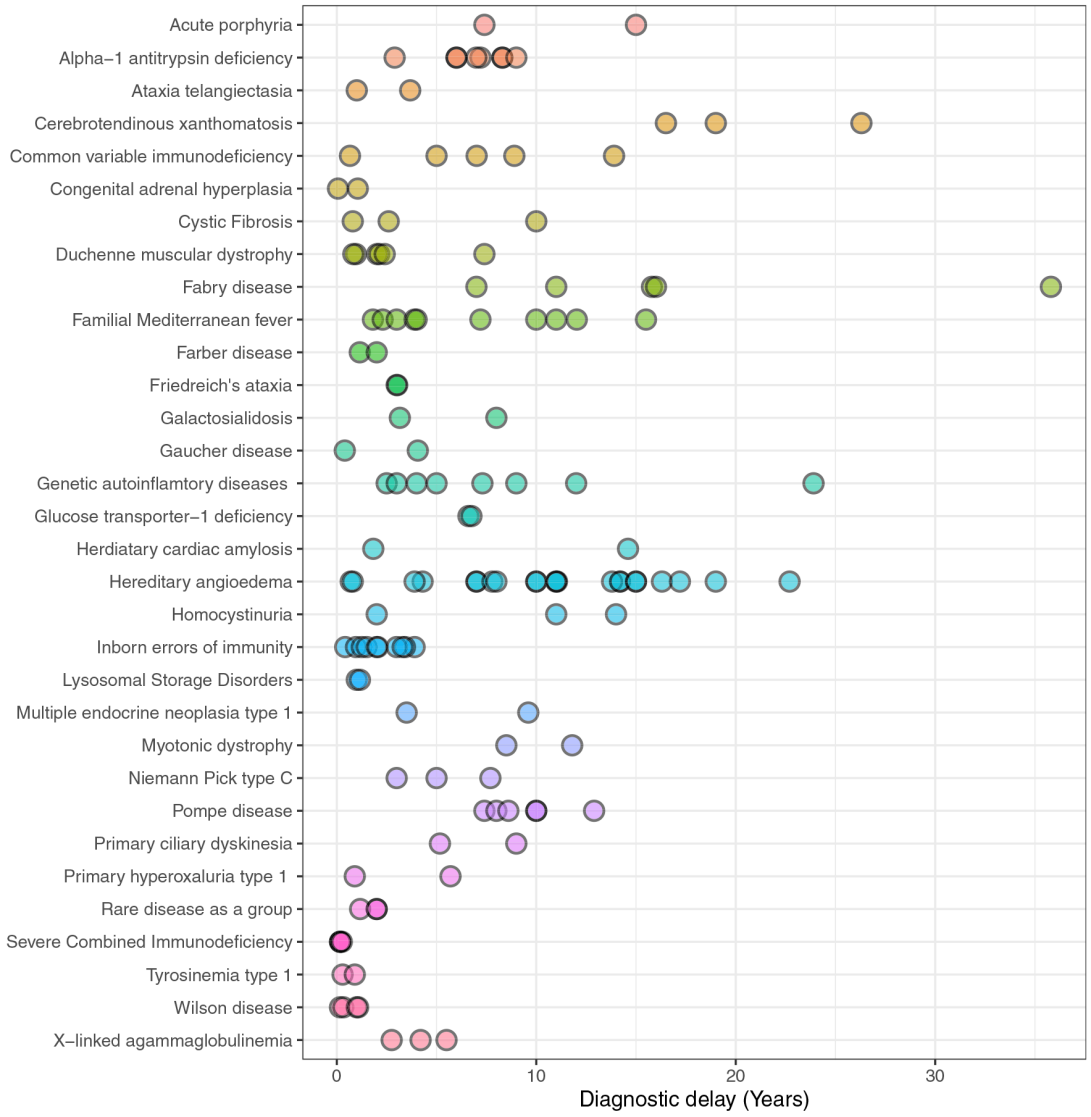
A figure demonstrating the range in the reporting of disease-specific diagnostic delay for all genetic disorders that have 2 or more studies.

For articles that reported disease specific diagnostic delay (*N* = 203/259), there is substantial variation between studies in the reporting of diagnostic delay (see Figure 3, Supplemental Table 4). For example, in hereditary angioedema, the disease with the most studies in our literature search, diagnostic delay ranged from 0.7 to 22.7 years. For genetic diseases that have 3 or more studies reporting diagnostic delay (*N* = 17), the median difference between the minimum and maximum reported diagnostic delay was 6.57 years (max to minimum range 0.09 to 28.8) (IQR 3.48-13.24) (see Supplemental Table 4).

### Diagnostic delay studies are not representative of genetic disease globally

**Figure 4:**
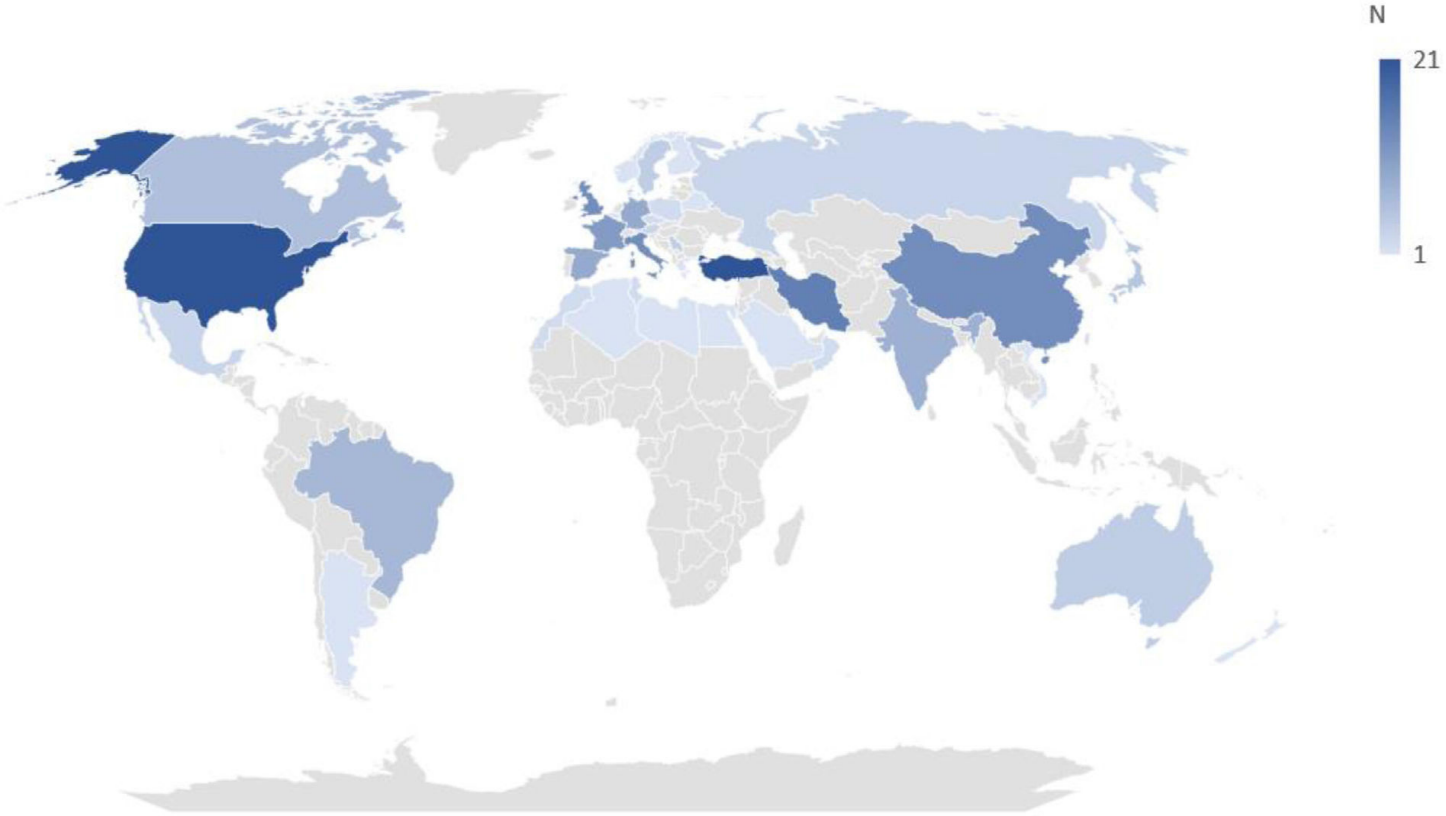
A geographical global heatmap demonstrating the most common countries where diagnostic delay length has been investigated in genetic disease.

Diagnostic delay studies have been conducted in 43 different countries (see Figure 4). The most frequent countries investigated in the literature were United States (*N* = 21), Turkey (*N* = 21), and Iran (*N* = 15). Forty-six of the 259 studies were multinational. There were no studies in the least developed countries (as defined by the United Nations). As per UN data of the 213 studies conducted in individual countries, 132 were conducted in the developed world, 81 in the developing world, and 0 in least developed countries (see Table 1 and Figure 4). For studies that reported diseases specific diagnostic delay developed countries (eg, the United States) reported a statistically significant longer diagnostic delay (Median 7.1 years) than developing countries (eg, Turkey and Iran) (median 2.99 years; *P* value <.01 unpaired *t* test).

### Early onset childhood disorders of metabolism, immunity and development are associated with shorter diagnostic delay

Of the 16 diseases with a reported diagnostic delay less than 1 year in the literature, 15 were early onset and 1 was late onset (see Supplemental Table 4). These were primarily disorders of metabolism (eg, maple syrup urine disease), immunity (eg, severe combined immunodeficiency), and development/regression (eg, Rett syndrome). Twenty-four genetic diseases reported a mean disease specific diagnostic delay of 10 years or greater. Of these 21 were classified as late onset disease. These were primarily adult onset: neurological disorders (eg, limb girdle muscular dystrophy) and cancer predisposition-overgrowth syndromes (eg, tuberous sclerosis). Our classification system of the age of onset genetic diseases can be found in Supplemental Table 4.

### There is limited consistency in diagnostic delay research methods

There is little consistency in the medical setting, diagnostic criteria, definitions used, and ascertainment in the diagnostic delay literature (see Table 1 and Supplemental Tables 5-7). There are 12 different definitions used to for diagnostic delay, 15 different types of study settings (eg, medical centers and patient support networks), and 18 different methods diagnostic delay ascertainment (Table 1, Supplemental Tables 5-7).

### Variations in the statistical methods used to report diagnostic affect its reported value

There is a lack of statistical standardization in the statistical reporting of results of studies investigating diagnostic delay. Of the 259 studies, we found 114 reported diagnostic delay with medians, 102 with means, 15 with both medians and means, 10 with range, 9 with averages (not specifying if this was mean or median), and no statistical reporting in 9 studies (only referring to the value of diagnostic delay). Of the 259 studies, 49 reported diagnostic delay as a split analysis and not collectively (eg, of different, phenotypes or genotypes). Studies reporting means had a significantly longer diagnostic delay compared with studies reporting median (7.1 years versus 3.0, respectively; *P* value < .01, Student *t* test)

### Diagnostic delay may be improving with time, but few interventions have been investigated

Thirty-seven of the 259 studies investigated if diagnostic delay improved with time. Of these, 25 (67.6%) had concluded that diagnostic delay had improved, whereas 12 (32.3%) did not. Of these 37 studies, 5 studies tested an intervention in improving diagnostic delay. The methods tested included the following: a computer algorithm, awareness campaign, the impact of the identification of a causative mutation, direct to consumer personal testing, and newborn screening. Two of these studies concluded that the intervention improved diagnostic (computer algorithm and the discovery of a causative mutation). One hundred twenty of the studies made recommendations on how to improve diagnostic delay (see Table 1), 44 studies suggest increasing physician awareness, 23 suggested more newborn screening, and 19 suggested improving health care systems. There were 7 other suggested interventions.

## Discussion

The current study attempts to systematically collect, describe, and analyze the literature that measures diagnostic delay. In this process we found that the number of diagnostic delay studies has grown rapidly during the last 10 to 15 years as genomic medicine has become established. Diagnostic delay research is global and occurs in 43 different countries and across 111 different genetic diseases. The research is varied in the methods of measuring diagnostic delay, the study setting used to do this, and the definition of diagnostic delay. Encouragingly, among the 37 studies that assessed changes in diagnostic delay over time, most found (68%) found that that diagnostic delay is improving over time. Only 5 studies have attempted to measure the impact of an intervention on diagnostic delay. This has resulted in limited data on efficacy of interventions to reduce diagnostic delay. Expanding the number of studies that investigate interventions in diagnostic delay could therefore allow the prioritization of which interventions are of most benefit to implement. Furthermore, despite the burden of genetic disease being global, diagnostic delay research is not globally equitable with no studies conducted in low-income countries. This is an important finding because low-income countries are often settings where individuals with treatable genetic disorders (eg, phenylketonuria) have the highest preventable mortality.^28,29^

Despite this increase in the quantity of academic output, there are multiple methodological and systemic issues with the literature that make it difficult to compare findings across studies. There is a vast lack of standardization in the process of measuring, reporting, defining, and analyzing diagnostic delay. Studies often use different definitions of diagnostic delay with different data ascertainments methods. We found wide variation within the reporting of diagnostic delay for the same genetic disease and some evidence that this can affect the reported diagnostic delay. Additionally, there is no consistency in the statistical reporting of diagnostic delay. Studies often report either mean or median and rarely both. This is a major problem in the literature, given we found that those who reported in mean (vs median) had a higher diagnostic delay. Furthermore, diagnostic delay research is not truly representative of genetic diseases based on their reported prevalence. When we compared the distribution of these studies with the prevalence of genetic disease, the most prevalent genetic diseases are not studied at the highest frequency. Part of the explanation could be that prevalent diseases with distinctive anatomic features or other pathognomonic findings visible in childhood (eg, Trisomy 21) may have little to no diagnostic delay. However, without systematic efforts to evaluate diagnostic delay across diseases, it is impossible to come to this conclusion for any “common” genetic diseases.

Diagnostic delay is not an intrinsic quality of a disease but rather an interaction between the disease and a health care context.^10^ The literature is then a product of the studies that attempt to measure this interaction.^10^ We found evidence that the health care system in which individuals with a genetic disease interacts with can affect diagnostic delay. Specifically, we found differences in diagnostic delay in the developed vs developing world. We also found that there are disease specific patterns to diagnostic delay, with diseases that are classically associated with early life onset having reduced diagnostic delay. When all our findings are taken together, our results suggest that diagnostic delay is a multifactorial product of the disease studied, the health care system it exists in, and the study design.

Given the vast variation in disease specific reporting of diagnostic delay, there is a real risk that the literature reflects study design as opposed to the diseases or health care system. This could therefore result in diagnostic delay being difficult to compare between studies. To overcome these systemic issues in the literature, we propose that diagnostic delay be measured within the concept of a common model (as has been suggested by previous authors).^30^ Studies could report the date of the first documented: patient reported symptoms, clinical suspicion, clinical diagnosis, genetic diagnosis, and treatment. Subintervals could then be calculated. This model could then be applied at scale measuring multiple genetic diseases in local health care systems or on large data sets (eg, all of US federalized electronic health records [EHR] data). Such an effort could allow for a data-driven approach to identify diseases most likely to be affected by diagnostic delay and opportunity to identify the factors that could be intervened on to reduce it.

## Conclusion

Research into diagnostic delay has accelerated over the last decade. Encouragingly, there is some evidence that diagnostic delay is being reduced with time but not evidence as to why this is happening. We identified several gaps in the literature: first, the evidence for diagnostic delay is limited to high- and middle-income countries with no retrieved studies conducted in low-income countries. Second, many genetic diseases, even higher prevalent diseases, are not yet represented in the literature. Finally, large differences in the measurement standards and study designs prevent comparison of delay between studies, which inhibit a comprehensive understanding of the problem and efforts to create systematic solutions.

## Supplementary Material

supplemental material

## Figures and Tables

**Table 1:** A summary table of the features of the diagnostic delay literature in genetic disease

Characteristics of the Diagnostic Delay Literature	Findings (*N*)
Top 10 genetic diseases investigated	Hereditary angioedema 26
Full list in the Supplemental Table 1 for other diseases	Familial Mediterranean fever 16 Inborn errors of immunity 12 Genetic autoinflammatory diseases 11 Alpha-1 antitrypsin deficiency 9 Pompe disease 8 Fabry disease 8 Duchenne muscular dystrophy 7 Severe Combined Immunodeficiency 6 Wilson disease 6
Number of patients in the studies	3-18182 (range) 446 (mean) 68.5 (median)
Top 10 countries investigated in diagnostic delay literature.	Multinational 46 Turkey 21
Full list in the Supplemental Table 1	USA 21 Iran 15 UK 13 China 13 Italy 13 France 11 Spain 9 Germany 9
Economic development status of the countries as per OECD data	Multinational 46 Developed 132 Developing 81 Least economically developed 0
Study settings	Singel academic medical center 124 National registry 41 Multiple academic medical centers 37 International registry 18 Literature 14 Patient support group 9 National lab 4 Commercial Study 3 Newborn screening network 2 Billing data 2 Drug outcome survey 1 Not specified 1 National network 1 Diagnostic lab 1 National biobank 1
Diagnostic delay ascertainment method	Retrospective chart review 135 Patient questionnaire 35 Clinical assessment 28 Physician questionnaire 19 Literature 13 Retrospective chart review plus questionnaire 8 Not mentioned 5 EMR records 2 Patient and physician survey 2 Retrospective chart review + literature 2 Billing data 2 Clinical assessment and chart review 2 Biobank data 1 Multiple registries 1 Literature and clinical assessment 1 Patient assessment and interview 1 Prospective data collection 1 Patient questionnaire and assessment 1
Diagnostic criteria	Billed data 2 Biochemical 13 Biochemical and clinical 2 Biochemical and genetic 40 Clinical 46 Clinical, biochemical, and genetic 2 Clinical and genetic 10 Clinical or genetic 2 Demographic 12 Demographic and genetic 1 Genetic 41 Literature 3 Not reported 66 Self-diagnosed 3 Society criteria 12 Treated patients 4
Diagnostic delay definition	Symptom onset to diagnosis 226 Self-reported 8 Presentation to diagnosis 6 Birth to dx 4 Referral to diagnosis 4 Not reported 4 Literature 2 Billing event to diagnosis 1 Suspicion to diagnosis 1 Symptom onset to differential diagnosis 1 Disease related visit to diagnosis 1 Newborn screen date to diagnosis 1
Statistics to report diagnostic day	Median 114 Mean 102 Mean and Median 15 Not reported 9 Range 10 Average 9
Studies that reported disease specific diagnostic delay value	203
Studies that reported disease specific diagnostic delay as a range	7
Studies that report stratified sub analysis by characteristics of disease (eg, phenotype or genotype).	49
Studies that measured diagnostic delay over time	37
Studies that reported that diagnostic delay improved with time	25
Studies that tested an intervention	5
Studies suggested intervention. Improved diagnostic delay	2
Interventions suggested	NONE 139 Awareness 44 NBS 23 Systems changes to clinical practice 19 More research 14 More genetic testing 10 Test family members 3 Multiple 3 Improved biochemical diagnosis 2 Algorithms 1 Study delay more as a concept 1

## Data Availability

All data used in this analysis are available in Supplemental Table 1, which includes annotated row level data of all studies analyzed.
