# Supplementary material for: Diagnostic delay in monogenic disease: A scoping review
Source: Genet Med. Author manuscript; Available in PMC 2024 May 31. (PMC11140588; doi:10.1016/j.gim.2024.101074)
Supplement: supplemental material [file NIHMS1994306-supplement-supplemental_material.zip › 1-s2.0-S1098360024000078-mmc3.pdf]

Figure 1

Search Strategy - ("Rare Diseases"[Mesh] OR "rare disease*"[tiab] OR "unusual disease*"[tiab] OR "orphan disease*"[tiab] OR "uncommon disease*"[tiab] OR "Genetic Diseases, Inborn"[Mesh] OR "inborn genetic disease*"[tiab] OR "genetic disease*"[tiab] OR "genetic disorder*"[tiab] OR "hereditary disease*"[tiab] OR "hereditary disorder*"[tiab] OR "hereditary illness*"[tiab] OR "single gene defect*"[tiab] OR "single-gene defect*"[tiab] OR monogenic[tiab] OR mendelian[tiab] OR "genetic affliction*"[tiab] OR "genetic defect*"[tiab] OR "genetic illness*"[tiab] OR "genetic syndrome*"[tiab] OR "hereditary affliction*"[tiab] OR "hereditary condition*"[tiab] OR "hereditary defect*"[tiab] OR "heredodegenerative disease*"[tiab] OR "inherited affliction*"[tiab] OR "inherited disease*"[tiab] OR "inherited disorder*"[tiab] OR "inherited illness*"[tiab]) AND ("Delayed Diagnosis"[Mesh] OR "delayed diagnos*"[tiab] OR "late diagnos*"[tiab] OR "diagnostic delay*"[tiab] OR "diagnosis delay*"[tiab])
